# Laparoscopic Appendectomy for Perforated Appendicitis Caused by a Fish Bone: A Case Report

**DOI:** 10.7759/cureus.80754

**Published:** 2025-03-18

**Authors:** Michinari Suzuki, Hiroshi Kondou

**Affiliations:** 1 Surgical Gastroenterology, Shunan City Shinnanyo Hospital, Shunan, JPN

**Keywords:** acute appendicitis, fish bone, foreign body, laparoscopic appendectomy, multiplanar reconstruction (mpr), surgical abdominal emergency

## Abstract

Foreign body ingestion is relatively common, with most objects passing through the gastrointestinal tract without complications. However, some foreign bodies can cause gastrointestinal perforation, leading to severe complications. Fish bone-induced gastrointestinal perforation is relatively common in regions with high fish consumption, but appendiceal perforation remains extremely rare and diagnostically challenging. We report a case of a 76-year-old male patient who presented with worsening lower abdominal pain. Computed tomography (CT) revealed a swollen appendix (16 mm) with a high-density area and surrounding fat stranding, confirming acute appendicitis caused by appendicolith. Emergency laparoscopic appendectomy revealed a markedly swollen appendix with a central perforation. A 10-mm wedge-shaped fish bone was identified within the appendiceal lumen, confirming fish bone-induced perforated appendicitis. Histopathological examination confirmed severe inflammation with focal necrosis. Postoperative multiplanar reconstruction (MPR) images revealed a wedge-shaped hyperdense linear structure within the appendix, which was identified as a fish bone. Fish bone-induced perforated appendicitis is a rare but important differential diagnosis in regions with high fish consumption. A detailed dietary history and careful review of MPR images on CT are essential for accurate preoperative diagnosis. Early recognition and laparoscopic surgical intervention can prevent severe complications and improve patient outcomes.

## Introduction

Most ingested foreign bodies pass naturally through the gastrointestinal tract; however, complications occur in less than 1% of cases [1,2]. In rare instances, foreign bodies cause gastrointestinal perforation, necessitating medical intervention. Fish bone-induced gastrointestinal perforation is relatively common in regions with high fish consumption, such as Japan. However, appendiceal perforation remains rare, as reported in previous studies [3-5]. This condition can lead to severe complications such as generalized peritonitis and abscess formation, highlighting the need for prompt diagnosis and treatment. We report a case of laparoscopic appendectomy for fish bone-induced perforated appendicitis. A detailed history of fish consumption and the use of multiplanar reconstruction (MPR) images on computed tomography (CT) were key factors in achieving an accurate diagnosis.

## Case presentation

**Figure 1:**
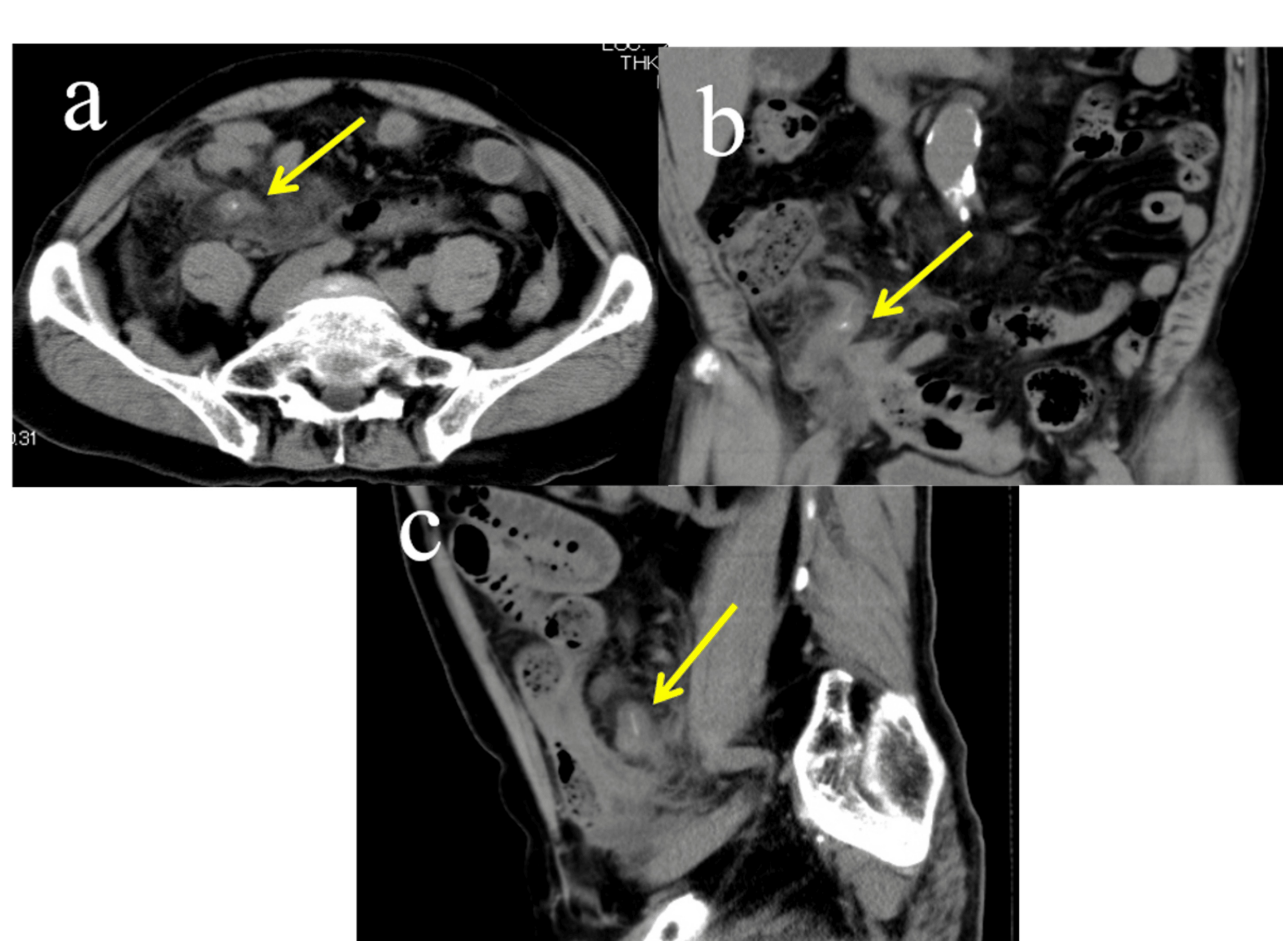
Computed tomography (CT) images of the abdomen. (a) Axial view. (b) Coronal view. (c) Sagittal view. The appendix was swollen to 16 mm in diameter, with a hyperdense shadow (yellow arrow) observed within, confirming acute appendicitis with appendicolith. Significant inflammatory spread to the surrounding tissues was noted, but no abscess formation was identified.

A 76-year-old male patient experienced lower abdominal pain that began the previous day and worsened overnight, prompting him to visit the emergency department. Upon arrival, his blood pressure was 128/68 mmHg, pulse rate 90 beats per minute, and body temperature 36.8 °C. Tenderness and mild muscular guarding were noted in the right lower abdomen; however, rebound tenderness was not observed. Blood biochemical tests revealed an elevated inflammatory response, with a white blood cell (WBC) count of 9,000/mm³ and a C-reactive protein (CRP) level of 8.0 mg/dL. However, no other abnormalities were detected in the laboratory findings. Abdominal X-ray revealed scattered small bowel gas but showed no evidence of air-fluid levels or free air. An abdominal CT scan demonstrated a hyperdense shadow within the appendix, which was swollen to 16 mm in diameter (Figure 1). Increased attenuation of the surrounding fatty tissue suggested the spread of inflammation. No obvious abscess formation was observed. Based on these findings, acute appendicitis with appendicolith was diagnosed. The preoperative differential diagnoses included cecal diverticulitis, Meckel's diverticulitis, terminal ileitis, and acute appendicitis caused by a fecalith. However, the absence of cecal or ileal enlargement on CT, along with the presence of an enlarged appendix and a high-density shadow within it, ultimately ruled out the first three conditions, leaving only the possibility of acute appendicitis caused by a fecalith. An emergency laparoscopic appendectomy was performed.

Intraoperative findings

**Figure 2:**
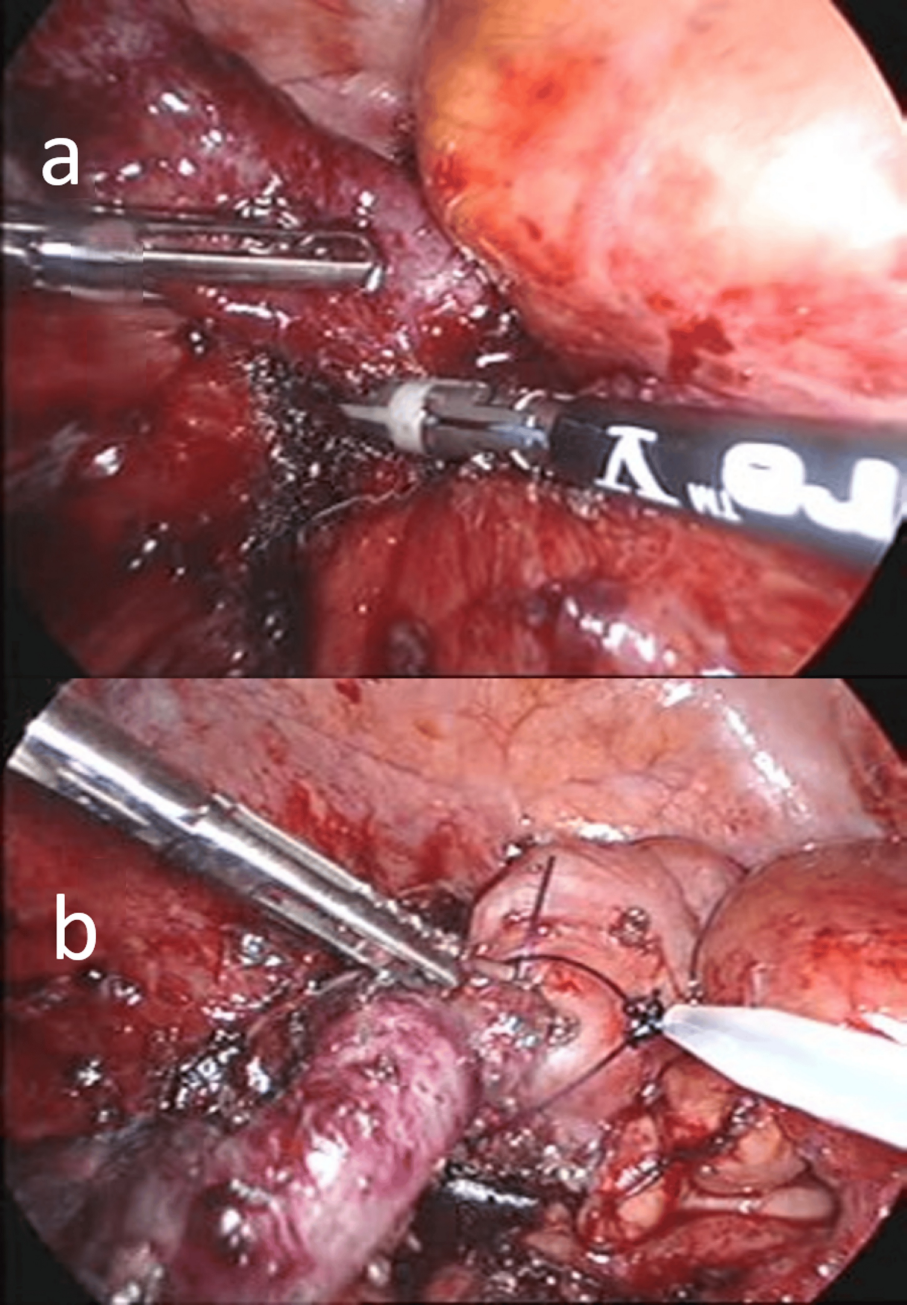
Intraoperative images of laparoscopic appendectomy. (a) The appendix was markedly swollen. As it was adherent to the ileum and greater omentum, laparoscopic adhesiolysis was performed. (b) The appendix was ligated using a pre-tied ligature loop device and transected.

**Figure 3:**
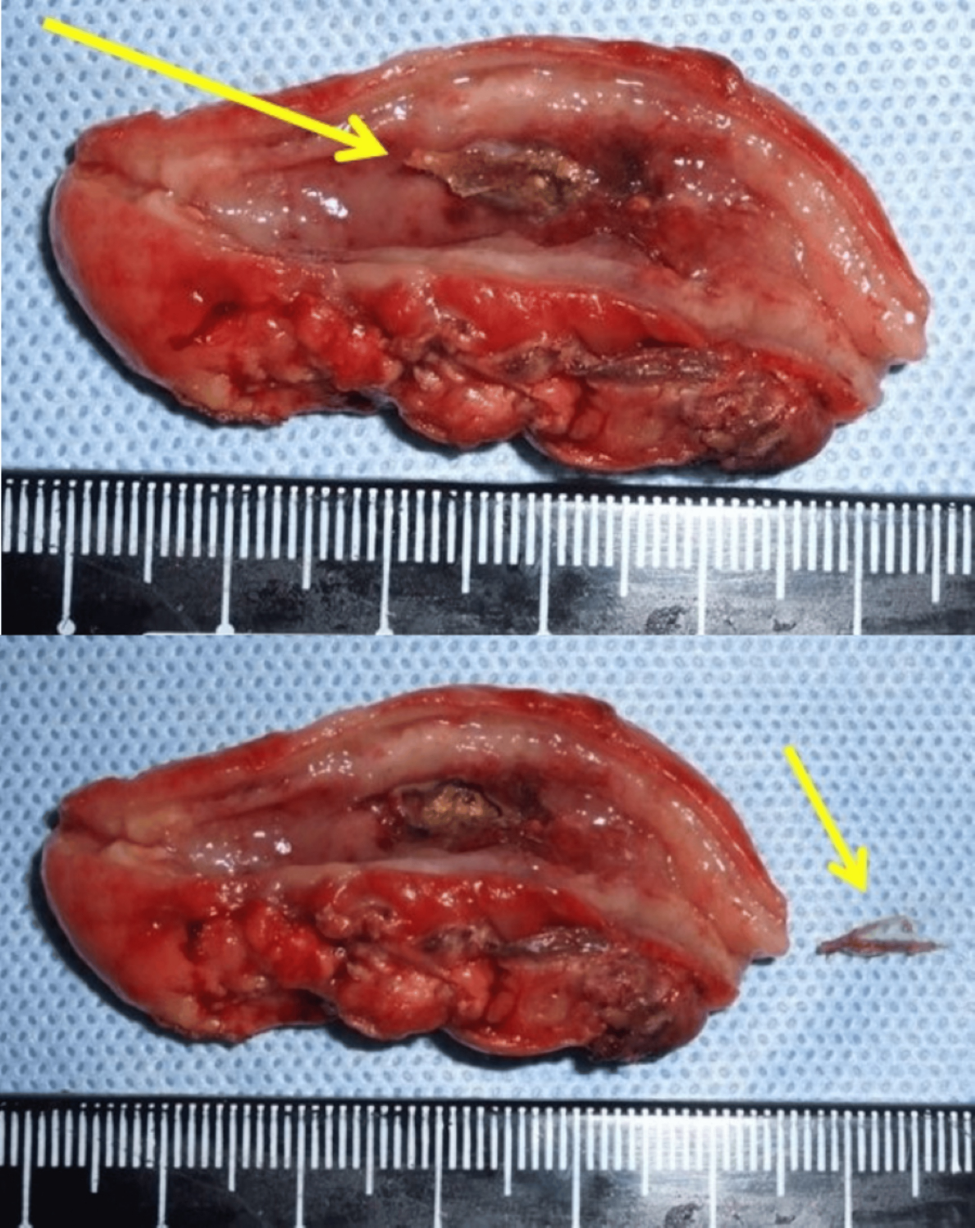
Gross specimen images of the appendix and embedded fish bone. The appendiceal wall was diffusely thickened, with marked mucosal erythema and edema. A 10-mm-long wedge-shaped fish bone (yellow arrow) was embedded in the central portion of the appendix, resulting in perforation.

The appendix was markedly swollen and adherent to the ileum and greater omentum. Pus accumulation was observed in the Douglas pouch. Upon adhesiolysis (Figure 2a), a perforation was identified in the central portion of the appendix, with pus and fecalith discharge from the site. The appendix was ligated using a pre-tied ligature loop device (Figure 2b) and transected, followed by the extraction of the appendix. The abdominal cavity was irrigated with 6 L of warm saline, and a Penrose drain was placed in the Douglas pouch and the right paracolic gutter. The total operative time was 95 minutes. Upon examination of the resected specimen, the appendiceal wall was diffusely thickened, with marked mucosal erythema and edema. A 10-mm-long wedge-shaped fish bone was embedded in the central portion of the appendix, resulting in perforation (Figure 3). The fish bone was presumed to originate from the base of a dorsal fin.

Pathological findings

The appendiceal wall exhibited prominent hyperemia with extensive neutrophilic infiltration throughout all layers. An ulcer was observed in the central region, accompanied by full-thickness necrosis.

Postoperative course

**Figure 4:**
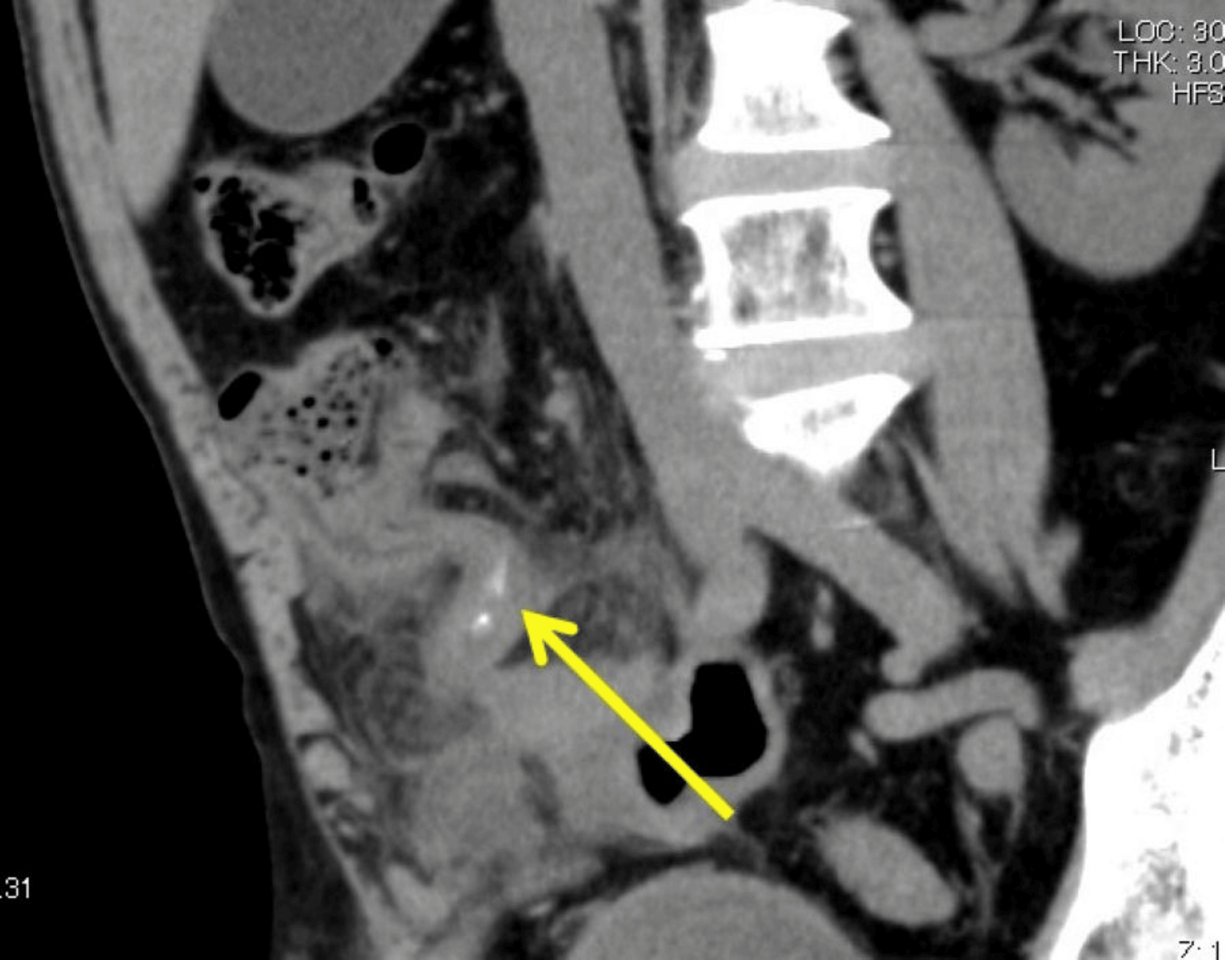
The multiplanar reconstruction (MPR) image from the abdominal CT scan. Postoperative MPR images reconstructed along the longitudinal axis of the appendix revealed a wedge-shaped hyperdense linear structure (yellow arrow) within the appendix, which was identified as a fish bone. The tip of the fish bone appeared to penetrate the appendiceal wall.

Postoperative MPR image reconstruction revealed a wedge-shaped hyperdense linear structure within the appendix, which was identified as a fish bone (Figure 4). A detailed postoperative history revealed that the patient enjoyed fish and had consumed a large rockfish a few days prior. It was presumed that the fish bone had been accidentally ingested at that time. Although the patient developed mild paralytic ileus postoperatively, there were no signs of wound infection or intra-abdominal abscess. Oral intake was initiated on postoperative day 5, and the patient was discharged in good condition on day 11.

## Discussion

Foreign body ingestion is relatively common; however, in most cases, the ingested object passes through the gastrointestinal tract without complications. The reported incidence of associated complications is less than 1% [1]. Fish bones and chicken bones are the most common culprits, followed by sharp objects such as toothpicks, needles, and thumbtacks. Among these, gastrointestinal perforation occurs in approximately 1% of cases and can lead to severe complications such as abscess formation, obstruction, and, in severe cases, generalized peritonitis, sepsis, and multiple organ failure [2,6]. Thus, prompt diagnosis and management are crucial. The terminal ileum and the duodenal C-loop are the most common sites of perforation caused by sharp foreign objects [7,8].

Appendicitis caused by foreign bodies is rare, with an estimated incidence of 0.005%-0.113% in large case series [3,4,9,10]. Perforated appendicitis due to fish bones is even rarer, estimated at less than 0.0005% [3-5]. However, as demonstrated in this case, fish bones can migrate into the appendix, causing inflammation and perforation. In regions with high fish consumption, clinicians should consider this possibility when evaluating patients with acute appendicitis.

The appendix is particularly susceptible to the accumulation of foreign bodies due to gravity. Once a foreign object enters the appendix, peristaltic movement alone is often insufficient for expulsion. Blunt objects may obstruct the lumen, leading to inflammation, whereas sharp objects can perforate the appendiceal wall, potentially causing peritonitis or periappendiceal abscess formation [11,12].

Detecting fish bones on plain abdominal X-rays is challenging, with a reported sensitivity of only 32% [13,14]. In contrast, CT is highly effective, with a sensitivity of 100% and specificity of 97.8%, for detecting fish bones [15]. Fish bones typically appear as linear calcified foreign bodies associated with localized inflammation [5,15], and direct visualization of calcification is crucial for definitive diagnosis [16].

In this case, CT imaging revealed a hyperdense linear shadow within the appendix. However, because fish bones had not previously been encountered as a causative agent, the shadow was initially misinterpreted as an appendicolith. Postoperative MPR images along the longitudinal axis of the appendix revealed a wedge-shaped hyperdense shadow, confirming the diagnosis of a fish bone. Goh et al. reported that the initial detection rate of fish bones on abdominal CT was 71.4% (five out of seven cases), but subsequent reevaluation improved the detection rate to 100% (seven out of seven cases) [16]. This highlights the importance of a careful review of CT images and the utility of MPR reconstruction for identifying foreign bodies [17].

Preoperative diagnosis of fish bone ingestion is often difficult because patients frequently do not recall swallowing fish bones. In this case, the patient remembered consuming fish but was unaware of ingesting a bone. Due to their small size, fish bones often pass asymptomatically and cause nonspecific symptoms, making preoperative diagnosis challenging. Therefore, obtaining a detailed dietary history and considering foreign body ingestion as a potential cause of acute appendicitis are essential [18].

Laparoscopic surgery is a minimally invasive approach that allows for detailed exploration of the affected area, safe dissection of adhesions, and thorough irrigation of the peritoneal cavity under direct visualization [19]. In this case, laparoscopic appendectomy facilitated adequate observation, adhesion dissection, and complete peritoneal lavage, potentially preventing postoperative complications. Given the diagnostic challenge of fish bone-induced appendicitis, laparoscopic surgery remains a valuable treatment option.

## Conclusions

Fish bone-induced perforated appendicitis is an extremely rare and diagnostically challenging condition. This case highlights the importance of considering foreign body ingestion in acute appendicitis, particularly in regions with high fish consumption. A thorough dietary history and the use of MPR images on CT can aid in accurate preoperative diagnosis, facilitating timely and appropriate management.
